# *Streptococcus agalactiae* prevalence and antimicrobial susceptibility pattern in vaginal and anorectal swabs of pregnant women at a tertiary hospital in Cameroon

**DOI:** 10.1186/s13104-018-3589-x

**Published:** 2018-07-16

**Authors:** Nkembe Marius Nkembe, Hortense Gonsu Kamga, Williams Abange Baiye, Anicette Betbui Chafa, Philip Nana Njotang

**Affiliations:** 1 Faculty of Medicine and Biomedical Sciences, University of Yaoundé 1, Yaoundé, Cameroon; 2 Franciscan Catholic Health Centre Mayo Darlé, Mayo Darlé, Adamawa Region Cameroon; 3 Department of Microbiology-Parasitology-Haematology-Immunology, Faculty of Medicine and Biomedical Sciences, University of Yaoundé 1, Yaounde, Cameroon; 4 Bacteriology Laboratory, University Teaching Hospital, Yaoundé, Cameroon; 5 Department of Obstetrics and Gynaecology, Faculty of Medicine and Biomedical Sciences, University of Yaoundé 1, Yaoundé, Cameroon; 6 Obstetrics and Gynaecology Unit, Yaoundé Central Hospital, Yaoundé, Cameroon

**Keywords:** Group B *Streptococcus*, Prevalence, Antimicrobial susceptibility, Pregnant women

## Abstract

**Objective:**

Group B Streptococcus (GBS) or *Streptococcus agalactiae* is part of the normal flora of the gut and genital tract, thus carrier pregnant women can transmit this germ to newborns which could cause early neonatal infection. In Cameroon, few studies have been conducted on GBS, thus this study sought to detect the rectal and vaginal colonization rates and the antibiotic susceptibility profile of the identified strains in pregnant women. We therefore conducted a cross-sectional study over a 6 months period analysing vaginal and anorectal samples obtained from 100 pregnant women. Cultures for the isolation of GBS were carried out according to standard microbiological methods and grouping done using the Pastorex strep Kit. All strains isolated were used for susceptibility test to various antibiotics as recommended by the French microbiology society, using the disk-diffusion method.

**Results:**

The detected colonization rate was 14%. No resistance to ampicillin, oxacillin, amoxycillin–clavulanate, cefotaxime, pristinamycin, vancomycin and clindamycin was found. Just 12, 94 and 82% of strains showed sensitivity to gentamycin, erythromycin and cefoxitin respectively. This study therefore revealed that at least one out of every ten women is GBS colonized and strains showed uniform sensitivity to beta lactamines. However, decreased sensitivity to erythromycin was detected.

## Introduction

The third sustainable development goal has as one of its targets to end preventable deaths in new-borns, countries aiming to reduce neonatal mortality to 12/1000 live births by the year 2030 [1]. In Cameroon neonatal mortality rate was 28/1000 live births in 2013 with the major causes being neonatal sepsis, prematurity and birth asphyxia [2]. Neonatal infection thus remains a chief cause of neonatal mortality in Cameroon, and GBS represents the second group of germs incriminated [3]. GBS is a harmless commensal bacterium being part of the gastrointestinal and genitourinary tracts of up to 30% of healthy human adults [4]. Hence an asymptomatic pregnant woman can transmit the germ to her foetus through the intraamniotic route and during passage through the birth canal [5]. The Centre for Disease Control and Prevention (CDC) recommends that vaginal and rectal GBS cultures be done on all pregnant women between 35 and 37 weeks gestation. They equally recommend intrapartum prophylaxis with Penicillin G to women with positive cultures, women with GBS bacteriuria and equally to women with a past history of an infant with invasive GBS disease [6]. Recommendations endorsed by the American College of Obstetricians Gynaecologists (ACOG) and American Academy of Paediatrics (AAP) in 2013.

Obtaining data on antibacterial susceptibility is essential to optimize treatment and minimize the emergence of bacterial resistance, which is responsible for the increasing number of therapeutic failure.

GBS colonisation rates among pregnant women in the world is 17.9%, with the highest rates recorded in Africa; 22.4% [7]. In Cameroon available data places this rate at 7.7% at the Yaoundé Gyneco-obstetric and Paediatric hospital [8] and at 6,7% at the Yaoundé General Hospital [9]. Although studies in Cameroon show a predominance of Gram negative bacterial sepsis among infants, contributing to infant mortality, it is possible that the role of GBS has been underestimated [3]. In Cameroon, infant mortality remains high at about 28/1000 live births [2], and few studies have been conducted on GBS neither are there public health policies aimed at the reduction of GBS neonatal infection, these deficits therefore prompted the following study.

## Main text

### Methods

We carried out a hospital-based cross-sectional study from 16th December 2016 to 15th May 2017, during which consecutive non-exhaustive sampling was used to recruit pregnant women in the third trimester. Excluded were HIV positive women, women currently on antibiotics, women with on-going gastroenteritis and those with haemorrhoids.

During routine ANCs, we selected clients eligible for the study, the study was explained to them and clients who accepted to take part then gave a written consent. Relevant information was collected using a questionnaire. While lying on the gynaecological bed, two samples were collected; one swab of the lower rectum and one of the lower vagina using sterile wooden cotton swabs. Each swab was then immersed into 3–5 ml of the Brain Heart Infusion Broth (BHIB), placed into the specimen transportation flask and conveyed to the lab within 30 min.

**Fig. 1:**
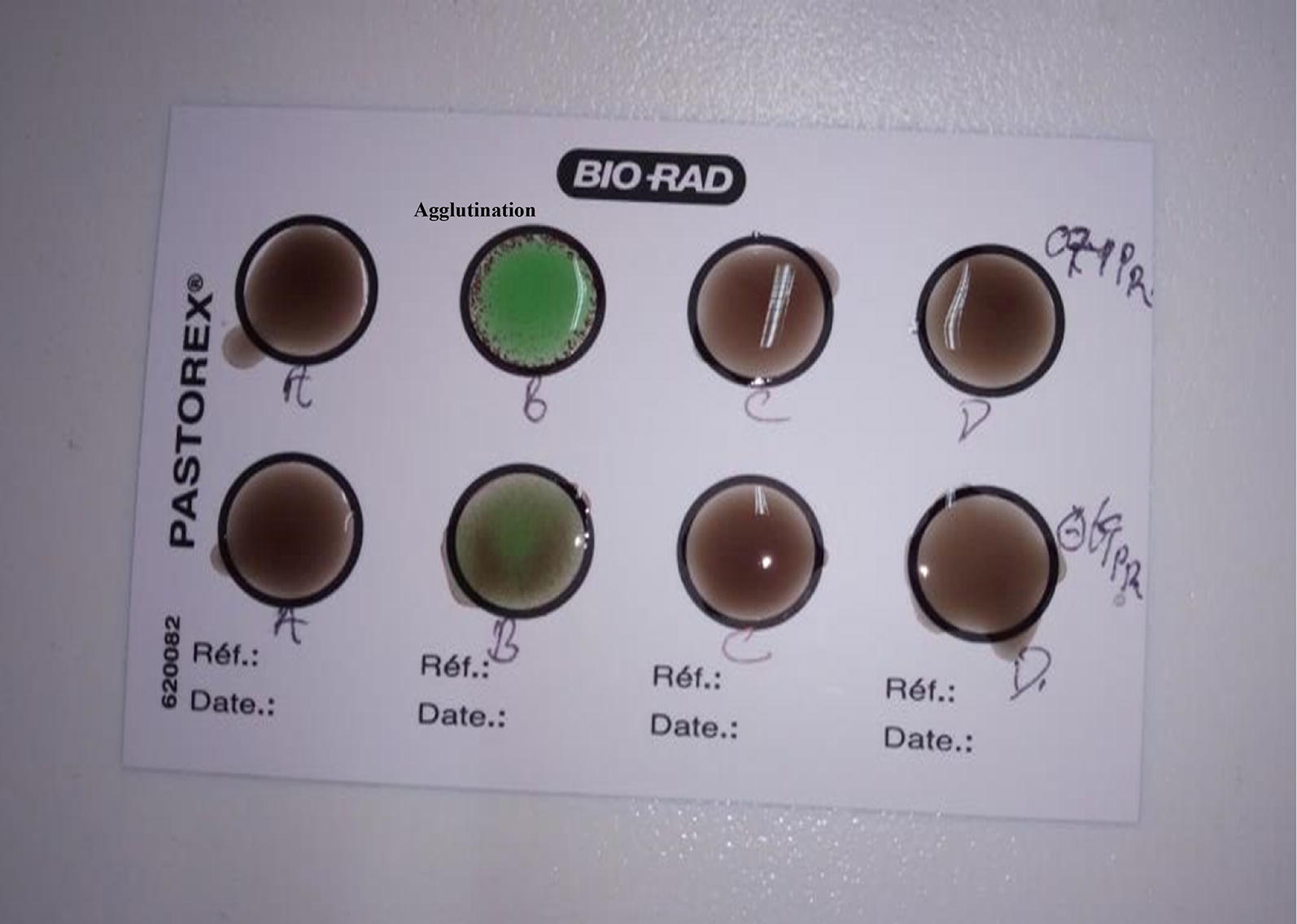
Agglutination results on the Pastorex card

Once in the lab, all specimens were inoculated into blood agar plates supplemented with colistin nalidixic acid and then incubated for 24 h at 37 °C in a jar enriched with 10% Carbon-dioxide. All greyish, smooth, small and non-pigmented colonies with a visible zone of beta hemolysis appearing 24 h after incubation were isolated, further incubated and their reactivity to catalase evaluated. Colonies with a negative catalase reactivity after further incubation were then isolated and used for the confirmatory diagnosis using the Pastorex strep kit (BIORAD). Colonies which only agglutinated with the GBS latex reagent were considered positive (Fig. 1). The resulting isolates were then used for antibiotic susceptibility testing by the Kirby Bauer disc diffusion method with the reference bacterial strain being ATCC 49619 *Streptococcus pneumonia*. The antibiotics tested and their respective diameters of inhibitions were those recommended by the French microbiology society as of 2016 (CASFM 2016).

After verifying and validating all information on the questionnaires and laboratory bench forms, all data were entered and analysed using EPI Info version seven. The major analysis included calculation of frequency and their confidence intervals at 95% (for qualitative variables), and mean or median (for quantitative variables).

### Results

A total of 100 pregnant women participated, hence 200 samples collected. The mean age of participants was 28.8 ± 5.4 years old, with the youngest being 18 and the oldest 44 years old. The various socio-demographic data are represented on Table 1.

**Table 1:** Representation of various socio-demographic factors *n *= 100

	Frequency	Percentage (%)
Age group (years)		
< 19	2	2.0
19 to < 24	14	14.0
24 to < 29	32	32.0
29 to < 34	33	33.0
34 to < 39	13	13.0
39 to < 44	5	5.0
44 to < 45	1	1.0
Educational level		
Illiterate	2	2.0
Primary	5	5.0
Secondary	46	46.0
Post-secondary	47	47.0
Occupation		
Civil servant	26	26.0
Farmer	1	1.0
House wife	18	18.0
Student	29	29.0
Trader	26	26.0
Gestational age group (weeks of amenorrhoea)		
28 to < 30	28	28.0
30 to < 32	11	11.0
32 to < 34	23	23.0
34 to < 36	9	9.0
36 to < 38	10	10.0
38 to < 40	12	12.0
40 to < 42	7	7.0
Gravidity		
Multigravid	70	70.0
Primigravid	30	30.0

GBS was isolated in the vagina and/or rectum of 14 participants giving us a prevalence of 14%. The rectal colonization rate was 13% while the vaginal colonization rate was 4%, hence a rectal to vaginal colonization ratio of about 3:1. A total of 17 GBS strains were isolated, with 10 isolated solely from rectal cultures (exclusive rectal colonization rate of 10%), one exclusively from vaginal cultures (exclusive vaginal colonization rate of 1%) and 6 concomitantly from vaginal and rectal cultures of the same woman (concomitant rectal and vaginal colonization rate of 3%). Therefore, isolated rectal colonization rate was 71.4% of overall carriage (10/14), isolated vaginal colonization 7.1% (1/14) of overall carriage while concurrent vaginal and rectal colonization was 21.4% (3/14) of overall carriage. Other germs isolated on vaginal cultures were *Candida albicans*, *Gardnerella vaginalis* and *Candida* spp. at 22, 18 and 16% frequencies respectively.

**Fig. 2:**
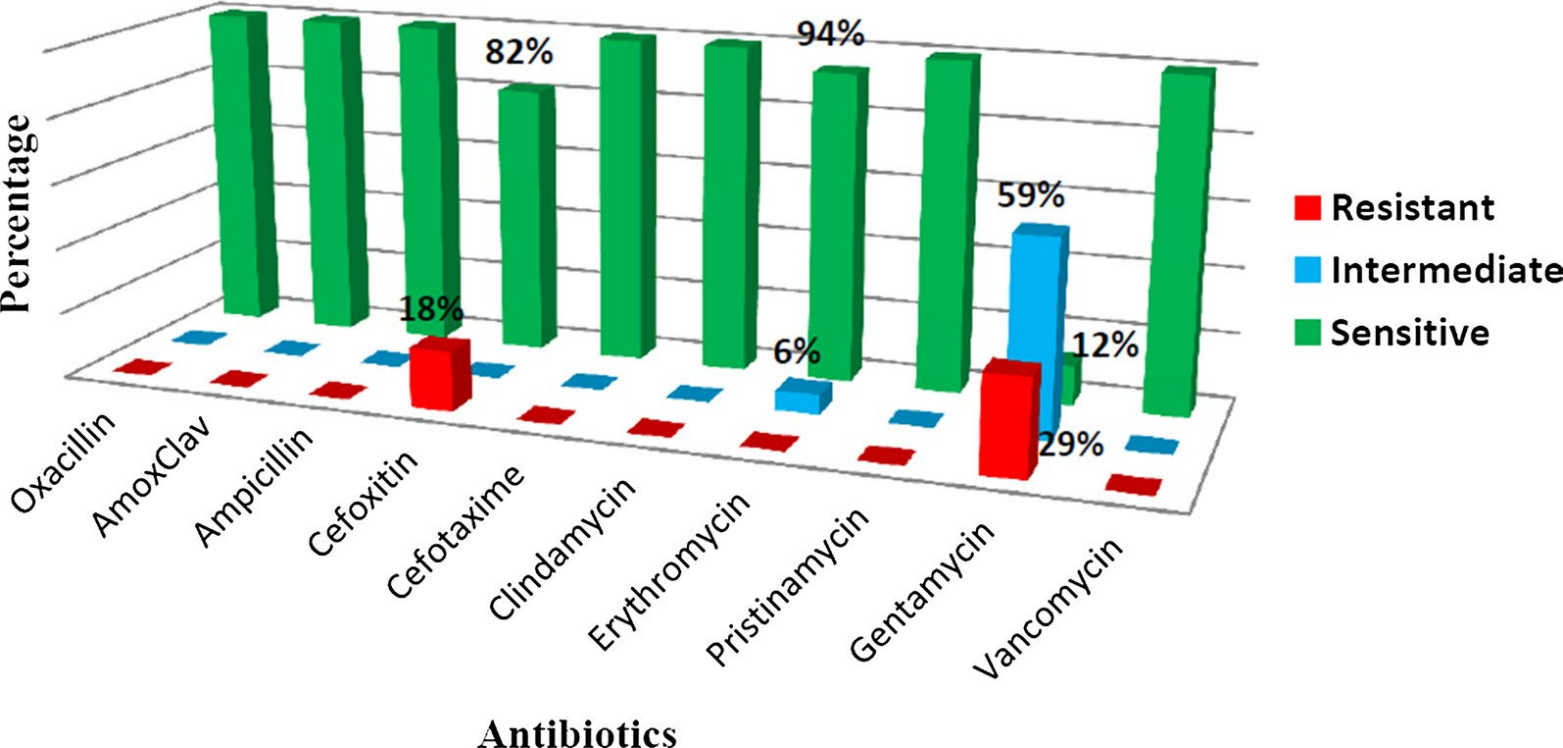
GBS antibiotic susceptibility profiles

The 17 GBS strains isolated were all sensitive to Penicillins (Oxacillin, amoxicillin–Clavulanate, Ampicillin), third generation cephalosporins (Cefotaxime), macrolides (Clindamycin and Pristinamycin) and glycopeptides (Vancomycin). However just 94.1% of the strains were sensitive to Erythromycin. About 58.8% of these strains had an intermediate sensitivity to Gentamycin with just 11.8% being susceptible (confer Fig. 2).

### Discussions

Our results revealed that most participants, 65% were between the mid-20s and early 30s, had completed at least basic and secondary and had at least been pregnant once (Multigravida). These results are different from those found by Turner et al. in 2012 in a similar study on a refugee population along the Thai-Myanmar border in South East Asia where the majority of carriers were in their 20s [10]. These results are however similar to those of Adawaye et al. in 2008 in Cameroon [8], those of Foumane et al. in Cameroon in 2002 [9] and those of Mengist et al. in Ethiopia in 2016 [11] where they respectively reported 75, 60 and 64% of participants to be in the above age range with 66.2, 69.6 and 70% being multigravida. These results can be explained by the fact that our study, as those of Adawaye, Foumane and Mengist took place in an urban setting where women turn to acquire a certain level of education before getting pregnant in contrast to the study carried out on the refugee population.

We found an overall colonization rate of 14%, with 4% vaginal colonization rate and 13% rectal colonization rate. These results are lower compared to 19% reported by Mengist et al. in Ethiopia in 2016 [11]; 20.5% reported by Mohamed et al. in Ethiopia in 2012 [12]; 20.2% by Bassir et al. in Marrakech in 2016 [13]; 18% by Ezeonu et al. in Nigeria in 2014 [14] and 16.5% by Dzowela et al. in Malawi in 2006 [15]. These rates are equally lower than those documented in a review by Kwatra et al. in 2016 where an overall rate of 17 and 22.4% was detected globally and for Africa respectively [7]. Other reviews revealed carriage rates of 20% in Nigeria, 19% in Cote d’ivoire, 4% in Togo, 20% in Gambia and 20% in Zimbabwe [16]. This disparity could be explained by the fact that, rates of GBS colonization vary widely throughout the world. Food habits, climate, maternal hygiene and culture methods, including the number and type of sites cultured and type of medium used, have accounted for some of these variations [17]. For example in our study, we collected lower vaginal and lower rectal swabs which is however in harmony with other studies, meanwhile other investigators collected upper vaginal swabs only and no rectal swab. We used the characteristic of the GBS colonies on blood agar plates with the agglutination test to determine carriage rate perhaps other investigators used other technics like PCR or hydrolysis of Hippuric acid [11]. This disparity equally proves the fact that GBS carriage rates vary per country as documented by Stoll et al. in 1998 [16]. This study equally revealed an isolated rectal carriage rate of 72%, 7% isolated vaginal carriage and a concomitant recto-vaginal colonisation rate of 21%. These were closer to results documented by Mengist et al. in Ethiopia in 2016 with predominant rectal carriage (46%) followed by 29% concomitant carriage and 25% vaginal carriage [11].

The prevalence in this study is a hospital prevalence and thus cannot be projected on the population in our region or country. However, this could be used as a base from which large scale epidemiological studies can be done to detect the actual carriage rate in our population.

Results of antibiotic susceptibility testing revealed all strains were sensitive to all penicillins tested (Oxacillin, Amoxicillin–Clavulanic acid and Ampicillin) and 3rd generation Cephalosporins tested (Cefotaxime). However highest level of resistance were recorded in the aminoglycoside antibiotic group (29% of strains resistant to Gentamycin and 59% with intermediate sensitivity); macrolide antibiotic group (6% of strains having an intermediate sensitivity to Erythromycin) and 2nd generation cephalosporin group, Cefoxitin (18% of strains were resistant). These results showed that beta-lactamines which constitute the recommended first and second line prophylaxis regimen [6] were all active on the isolated strains. However, Erythromycin which is recommended in case of allergy to beta-Lactamines was not active on some strains. Similar results were reported by Mengist et al. in Ethiopia in 2016 with 100% susceptibility of strains to Penicillin G and Amoxicillin and reduced susceptibility (90%) of the later to Erythromycin. Same results were found in Saudi Arabia by Khan et al. in 2015 with reduced susceptibility to macrolides, 16 and 5% resistance to Erythromycin and Clindamycin respectively [18]. Susceptibility of GBS to Gentamycin and Cefoxitin as detected by this study closely correlates with those reported by Adawaye et al. with 100% of strains resistant to Gentamycin and 82% resistance to Cefuroxime a 2nd generation cephalosporin. High resistance to aminoglycosides could be explained by the fact that these antibiotics have limited action on streptococcal bacteria.

### Conclusion

In a setting where routine third trimester screening for GBS is not a routine, talk less of intrapartum prophylaxis, we found the prevalence of GBS to be high among pregnant women. However, all strains isolated showed uniform sensitivity to beta lactamines which make up the first and second line prophylactic regimens. Nevertheless, there exist strains with reduced susceptibility to Erythromycin, a drug recommended for intrapartum prophylaxis in case of allergy to the penicillins. We therefore recommend a routine GBS screening in vaginal and especially rectal swabs of pregnant women in the third trimester in our setting. Given that our prevalence was hospital based, we equally recommend large scale epidemiological studies be done in other parts of the country to know the actual GBS colonisation rate so as to better orientate public health policies towards the implementation of strategies of prevention.

## Limitation

- We had a small sample size. This was due to the limited study period and limited budgeting. This could equally be explained by the fact that the proportion of pregnant women met in the third trimester during our study period was small.

## Abbreviations

AMR: antimicrobial resistance
ANC: antenatal consultation
ATC: American Type Culture Collection
CASFM: Comité d’Antibiogramme de la Société Française de Microbiologie
CDC: Centres for Disease Control and Prevention
GBS: group B *Streptococcus*
